# Impact of a Supervised Twelve-Week Combined Physical Training Program in Heart Failure Patients: A Randomized Trial

**DOI:** 10.1155/2019/1718281

**Published:** 2019-09-03

**Authors:** Tainá Fabri, Aparecida Maria Catai, Fábio H. O. Ribeiro, Jonas A. Araújo Junior, Juliana Milan-Mattos, Danielle A. A. Rossi, Regina C. Coneglian, Ricardo C. Borra, Silmeia Garcia Zanati Bazan, João Carlos Hueb, Beatriz Bojikian Matsubara, Meliza Goi Roscani

**Affiliations:** ^1^Internal Medical Department São Paulo State University (UNESP), Botucatu Medical School, Botucatu, São Paulo State, Brazil; ^2^Physical Therapy Department, São Carlos Federal University (UFSCar), São Carlos, São Paulo State, Brazil; ^3^Biosciences Department, São Carlos Federal University (UFSCar), São Carlos, São Paulo State, Brazil; ^4^Medical Department, São Carlos Federal University (UFSCar), São Carlos, São Paulo State, Brazil

## Abstract

**Purpose:**

The aim of this study was to compare the effects of supervised combined physical training and unsupervised physician-prescribed regular exercise on the functional capacity and quality of life of heart failure patients.

**Methods:**

This is a longitudinal prospective study composed of 28 consecutive heart failure with reduced ejection fraction patients randomly divided into two age- and gender-matched groups: trained group (*n* = 17) and nontrained group (*n* = 11). All patients were submitted to clinical evaluation, transthoracic echocardiography, the Cooper walk test, and a Quality of Life questionnaire before and after a 12-week study protocol. Categorical variables were expressed as proportions and compared with the chi-square test. Two-way ANOVA was performed to compare the continuous variables considering the cofactor groups and time of intervention, and Pearson correlation tests were conducted for the associations in the same group.

**Results:**

No significant differences between groups were found at baseline. At the end of the protocol, there were improvements in the functional capacity and ejection fraction of the trained group in relation to the nontrained group (*p* < 0.05). There was time and group interaction for improvement in the quality of life in the trained group.

**Conclusions:**

In patients with heart failure with reduced ejection fraction, supervised combined physical training improved exercise tolerance and quality of life compared with the unsupervised regular exercise prescribed in routine medical consultations. Left ventricular systolic function was improved with supervised physical training.

## 1. Introduction

Heart failure (HF) is a pathological state in which an abnormality of cardiac function is responsible for failure of the heart to pump blood at a rate commensurate with the requirements of the metabolizing tissues or to do so only from an elevated filling pressure [1].

Despite advances in the treatment of HF, its mortality and morbidity are still alarming [2]. In addition, both intolerance to exercise and recurrent hospitalizations of patients with HF compromise their working life and quality of life (QOL) [3].

It has already been established in the literature that regular physical exercise (PE) can act favorably in several aspects: higher exercise tolerance, improvement in NYHA functional class, increased oxygen consumption at peak and at anaerobic threshold, and quality of life [4–8]. However, the role of diastolic and systolic functions in the improvement in functional capacity (FC) and QOL in a regular PE program is a matter of controversy [1–16]. For example, according to Gary et al. [8], a combined PE program for 12 weeks, 3 times/week, was able to improve FC, skeletal muscle strength, severity of symptoms, and QOL in patients with HF. This improvement occurred independently of changes in cardiac function. Other mechanisms such as vasodilation promoted by nitric oxide synthesis and attenuation of inflammatory response may be involved [9].

Because of the beneficial effects of exercise on several aspects of heart failure outcome, it has been routinely prescribed to patients with HF in cardiology consultations [8–10]. However, an unsupervised regular exercise is the usual way of medical orientation in these cases because such programs are unassessable for most of the patients. This would be an advantage because the regularity and intensity of the PE are presumed and not proven. Therefore, we hypothesized that supervised combined physical training (CPT) including aerobic and resistance exercise would be associated with a more favorable impact on the QOL and FC of HF patients, compared with an unsupervised one. In addition, we also hypothesized that these effects would be associated with an improvement in cardiac function as assessed by transthoracic echocardiography.

The aim of this study was to compare the effects of a supervised CPT and unsupervised physician-prescribed regular exercise on the FC and QOL of patients with HF and reduced ejection fraction (HF_REF_) and to correlate these findings with clinical and echocardiographic variables.

## 2. Methods

**Figure 1:**
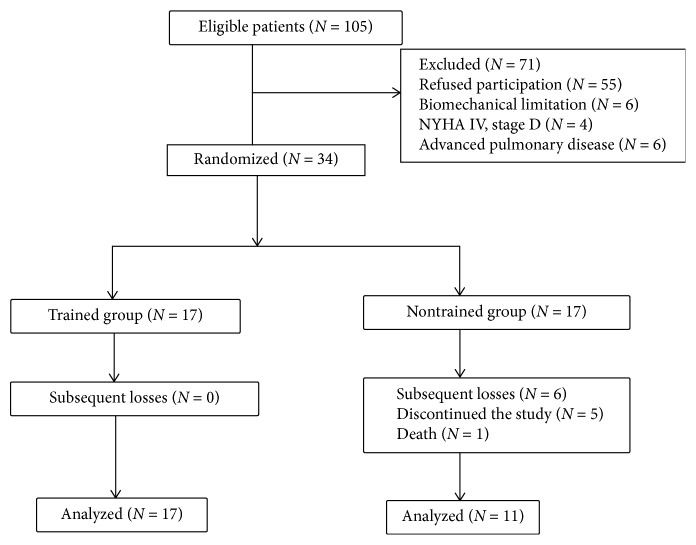
Flowchart showing the progression of patients in the study.

This study followed the criteria of the Consolidated Standards of Reporting Trials (CONSORT) statement, as reported in Figure 1. This is a randomized controlled trial including patients diagnosed with HF and left ventricular ejection fraction (LVEF) less than 50% and consecutively referred from cardiology outpatient clinics of University Hospital of Botucatu Medical School, São Paulo State University. The inclusion criteria were patients with HF with LVEF <50%, with optimized drug therapy and age over 50 years. The exclusion criteria were HF NYHA class IV and/or Stage D, decompensated HF in the last three months, atrial fibrillation and/or presence of an implantable device at the time of inclusion, chronic obstructive pulmonary disease (COPD), or biomechanical limitations to exercise.

The patients were divided into two groups matched for age and gender: trained group (TG) and nontrained group (NTG). Random allocation of patients was performed using computer-generated random numbers. Initially, 17 patients were included in each group; however, five patients of the NTG discontinued the study and one patient of the NTG died before undergoing the last evaluations. There was no loss to follow-up in the TG.

- TG, *n* = 17: all patients were submitted to 12 weeks of CPT supervised three times a week by the same physical educator. The CPT program consisted of moderate-intensity aerobic exercise (50 to 65% peak VO_2_), complemented by moderate-intensity resistance exercise (50% of the maximal voluntary contraction), which included the main muscle groups: biceps, triceps, quadriceps, hamstrings, and gastrocnemius. The prescription of CPT was individualized. The frequency considered as attendance of the supervised exercise sessions was 80%.
- NTG, *n* = 11: patients were prescribed only regular exercise, at least three times a week, according to American Society guidelines for HF^10^, and did not take part in the supervised CPT program.

This study was conducted in accordance with human research ethics standards and was approved by the Ethics Committee of São Paulo State University (UNESP) Medical School (protocol no. 19683313.5.0000.5411). The study was also registered in the ClinicalTrials.gov registry (number NCT02571270).

All patients were submitted to the following evaluations at the beginning and end of the protocol:

- Clinical evaluation includes age, gender, race, cardiovascular risk factors, NYHA functional class of HF, stage of disease, and physical examination in accordance with ACCF/AHA guidelines [10].
- Physical evaluation includes anthropometric measurements, body composition, and the Cooper walk test with measurement of relative oxygen consumption (VO_2_) and metabolic equivalents (METs) [11]. The examiner was blinded to the patient's group and the phase of the protocol.
- Transthoracic echocardiography evaluates systolic and diastolic function according to recent guidelines from the American Society of Echocardiography [12]. The examiner was blinded to the patient's group and the phase of the protocol.
- Quality of life assessment is performed using the “Medical Outcomes Study 36-Item Short-Form Health Survey” (SF-36) [13]. The patients answered to the questionnaire without intervention of the examiners.

### 2.1. Statistical Analysis

Continuous variables were expressed as means and standard deviations, or medians and interquartile ranges. Categorical variables were expressed as proportions and compared with the chi-square test. Two-way ANOVA was performed to compare the continuous variables considering the cofactor groups and time of intervention, and Pearson correlation tests were conducted for the associations in the same group. The level of significance was set at *p* < 0.05.

## 3. Results

The study included 17 patients in the TG and 11 patients in the NTG. The exercise of the TG was supervised by a physical educator in all patients, 3 times a week.  Table 1 shows the characteristics of the groups. There were no significant differences between the groups at baseline. The symptom of dyspnea improved in the TG at the end of the protocol (*p*=0.022). The groups were similar as to the prescribed drug therapy, which included angiotensin-converting enzyme (ACE) inhibitors (*p*=0.31), aldosterone inhibitors (*p*=0.61), angiotensin II receptor blockers (ARBs) (*p*=0.39), beta-blockers (*p*=0.33), digitalis (*p*=0.80), and diuretics (*p*=0.25).

Table 2 shows the morphological and functional echocardiographic variables before and after the protocol. The groups were similar at the beginning of the protocol. There was a significant improvement in the ejection fraction, assessed by Simpson's method, in the TG compared with that in the NTG at the end of the protocol (*p*=0.029). There was no change in diastolic function assessed by tissue Doppler imaging and left atrial volume index.

**Figure 2:**
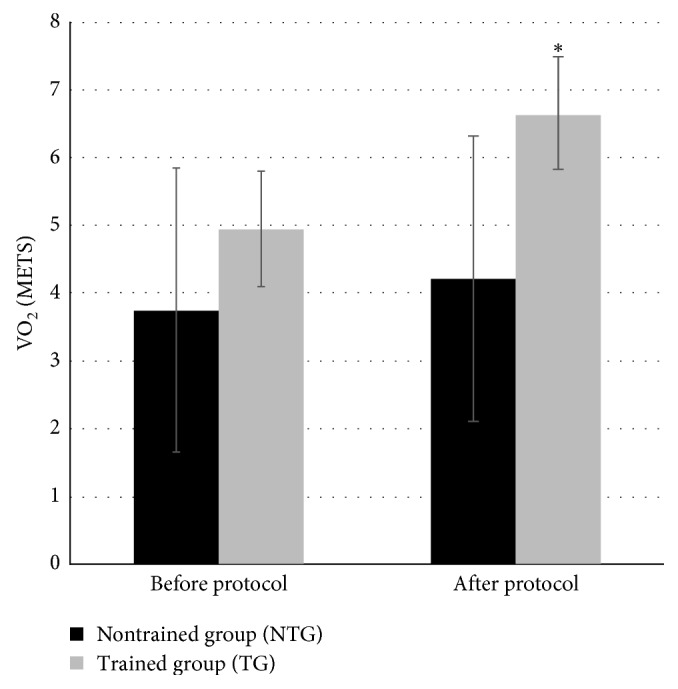
Comparison of peak VO_2_ between groups and time of the protocol. Values expressed in METS (metabolic equivalents) before and after the protocol. NTG = nontrained group; TG = trained group. There was no interaction between time and group (*p*=0.27). There was a significant difference between groups at the end of the protocol (^*∗*^*p*=0.002).

Figure 2 shows that there was a significant improvement in VO_2_ (METS) in the TG compared with that in the NTG at the end of the protocol (*p*=0.002). There was no interaction between time and group (*p*=0.27) for this increase in VO_2_.

The results of the QOL questionnaire are presented in Table 3. The analyses showed a significant interaction between time and group in the improved QOL of the TG in the following dimensions: physical functioning (*p* < 0.001), role-physical (*p*=0.017), vitality (*p*=0.011), and mental health (*p* < 0.001). There was a significant difference between groups at the end of the protocol in social functioning (*p* < 0.001) and role-emotional (*p* < 0.001). There was a difference in the time of the protocol in the TG in the dimension bodily pain (*p* < 0.001).

At the end of the protocol, the TG showed a significant association between FC, assessed by peak VO_2_ in METs, and the QOL questionnaire in the following dimensions: vitality (*R* = 0.5, *R*^2^ = 0.2; *p*=0.004), social functioning (*R* = 0.5, *R*^2^ = 0.3; *p*=0.002), and role-emotional (*R* = 0.6, *R*^2^ = 0.4; *p*=0.007).

There was no Pearson correlation with improvement in left ventricular systolic function and FC and QOL.

## 4. Discussion

Regular PE is beneficial for patients with HF_REF_. Although there is controversy regarding the mechanisms underlying the improvement in exercise tolerance and quality of life in these patients, our study has shown that CPT improved FC and QOL independently of any change in either left ventricular diastolic or systolic function.

At baseline, the TG and NTG were quite homogeneous, without significant differences in age, gender, ejection fraction, and optimized drug therapy.

Regarding the echocardiographic variables, only the LV ejection fraction in the TG showed a significant improvement at the end of the study. The mechanisms implied in this effect were not investigated in the present study. However, it is reasonable to assume that exercise-induced vasodilation might have a role by reducing the cardiac afterload and consequently increasing cardiac output and ejection fraction [9, 14–26]. Many other factors unrelated to exercise would also have interfered with this improvement, i.e., optimized HF medication, time of ischemia, and long-term revascularization procedures [22–25]. The small sample size did not allow us to determine the isolated influence of exercise on left ventricular systolic function. A recent meta-analysis showed that exercise training of short duration (<6 months) modestly increased LVEF, similarly to that presented in this study [27].

The mechanisms involved in the improvement in FC and QOL by exercise training are not well established. The available literature suggests that peripheral mechanisms, such as improved oxygen extraction in the active skeletal muscles, vasodilation promoted by increased nitric oxide synthesis, and attenuation of inflammatory response, may be responsible for greater exercise tolerance [15–17].

The significant improvement of subjective sensation of dyspnea in the TG (*p*=0.022) suggested a beneficial effect of CPT. Additionally, the social effect of the CPT probably contributed to the improvement in QOL and perception of symptoms.

HF patients present physical, emotional, and social impairments. The reasons [17–21] include significant loss of skeletal muscle mass, loss of independence in daily life, and intolerance to exercise. Physical rehabilitation programs are essential for bringing back, at least in part, the standard of living that the individual had prior to the event or diagnosis of HF.

The SF-36 questionnaire showed the improvement associated to CPT in the TG. All of the dimensions of the questionnaire showed significant improvement after the 12-week program, confirming the beneficial effect of CPT on QOL compared with the unsupervised regular exercise prescribed in cardiology consultations. This result is important because it strengthens the role of a multidisciplinary team in the care and monitoring of these patients.

The main limitation of this study was the small sample size. This is a known problem of interventional prospective studies. On the other hand, the important results we have shown, even in a small sample, rather reinforce the benefits of a supervised exercise program in HF_REF_ patients.

Another limitation of the study was the loss to follow-up of six patients in the NTG. Probably, one cause for the discontinuation of these patients was that the exercises were not supervised and the patients felt less motivation to participate in the study. Prescription of medication and change in life behavior were the same in both groups. Nevertheless, composition of a socialized exercise group may have influenced the nonwithdrawal of patients from the TG.

CPT improved exercise tolerance in the HF patients at the end of the protocol. In the present study, this improvement was not associated with any morphological or functional parameter of transthoracic echocardiography. These data are in agreement with those observed by others [16–19]. A recent meta-analysis [20] showed that exercise training improved fitness and quality of life in patients with HF even without significant echocardiographic changes. The findings suggest that exercise training can improve FC and QOL in patients with HF_REF_ independently of left ventricular diastolic function.

The positive association between FC and three dimensions of the QOL questionnaire (vitality, social functioning, and role-emotional) was more marked than in most previous studies [17–19] and suggests that the increase in exercise tolerance promoted by a supervised and individualized combined training is an important factor for improving the QOL in these patients.

## 5. Conclusion

In patients with HF_REF_, supervised CPT plays an important role in improving exercise tolerance and quality of life compared with the unsupervised regular exercise prescribed in routine medical consultations. We also found an improvement in systolic cardiac function as assessed by transthoracic echocardiography.

## Figures and Tables

**Table 1:** Baseline and clinical characteristics.

Characteristics (*n* = 41)	Nontrained group (*n* = 11)	Trained group (*n* = 17)	*p* ^*∗*^
Age (years)	61 ± 8	66 ± 10	0.26
Gender (M/F) (%)	45/55	75//25	0.09
Race (W/nW) (%)	50/50	69/31	0.28
SAH (%)	35	64	0.10
DM-2 (%)	35	45	0.31
Obesity (%)	15	27	0.36
Smoking (%)	45	27	0.12
Blood pressure			
Systolic (mmHg)	125 ± 22	127 ± 15	0.17
Diastolic (mmHg)	64 ± 12	62 ± 14	0.55
Heart rate (bpm)	73 ± 4	68 ± 3	0.16
BMI (kg/m^2^)	26.8	27.8	0.84

Values expressed as mean ± standard deviation or percentage (%) at the beginning of the protocol. M = male; F = female; W = white; NW = non-white; SAH = systemic arterial hypertension; DM-2 = diabetes mellitus type 2; BMI = body mass index; *p* = significance level; ^*∗*^<0.05.

**Table 2:** Morphofunctional echocardiographic variables.

Variables (*n* = 38)	Nontrained group (*n* = 11)		Trained group (*n* = 17)		*p* ^*∗*^
	Before	After	Before	After	
LVDD (mm)	61 ± 10	61 ± 8	60 ± 9	60 ± 8	0.84
LA (mm)	42 ± 6	40 ± 3	42 ± 11	43 ± 5	0.14
ILVM (g/m^2^)	234 ± 101	224 ± 66	176 ± 39	202 ± 72	0.46
EF (Simpson)	0.35 ± 0.1	0.34 ± 0.11	0.39 ± 0.1	0.44 ± 0.1^#^	0.45
ILAV (ml/m^2^)	27 ± 10	23 ± 8	31 ± 14	24 ± 7	0.66
*E*′ (cm/s)	7 ± 2	6 ± 2	7 ± 2	7 ± 4	0.40
*E*/*E*′	10 ± 3	12 ± 8	10 ± 5	11 ± 6	0.63

Values expressed in mean ± standard deviation before and after the protocol. LVDD = left ventricular diastolic diameter; LA = left atrium diameter; ILVM = indexed left ventricular mass; EF = ejection fraction by the Simpson method; ILAV = indexed left atrium volume; *E*′ = mitral tissue Doppler velocity (medium of lateral and septal velocities); *E* = wave velocity of mitral flow Doppler velocity; *p* = significance level of interaction between time and group (ANOVA); ^*∗*^<0.05; ^#^significant difference between groups.

**Table 3:** Comparison of the dimensions of the SF-36 questionnaire between groups.

SF-36 dimensions (*n* = 28)	Nontrained group (*n* = 11) Before/after	Trained group (*n* = 17) Before/after	*p*
Physical functioning	48 ± 11/53.6 ± 12	46.4 ± 15/80 ± 8.6	^*∗*^<0.001
Role-physical	49 ± 12.2/57.7 ± 14	50 ± 18/77.6 ± 10.3	^*∗*^<0.017
Vitality	50.9 ± 12.2/56.3 ± 12.8	52.9 ± 16/77 ± 9	^*∗*^0.011
Mental health	54.5 ± 16/56 ± 14.2	63 ± 11.5/75.8 ± 8	^*∗*^<0.001
Social functioning	53 ± 13/56 ± 15	70 ± 12/76.4 ± 11	^#^<0.001
Role-emotional	52 ± 13.2/55 ± 13	64 ± 15/78.2 ± 11	^#^<0.001
Bodily pain dimension	51 ± 14.4/61.3 ± 15	45.2 ± 22/77 ± 10	^&^<0.001
General health	57 ± 11/57.2 ± 12.7	49.4 ± 14/71.8 ± 8	0.12

Values expressed as mean ± standard deviation. Significant improvement in the trained group was observed by performing ANOVA, considering a significance level *p* < 0.05. ^*∗*^The difference between interaction of time and group, in the following dimensions: physical functioning, role-physical, vitality, and mental health. ^#^Significant difference between groups at the end of the protocol in social functioning and role-emotional. ^&^Difference in the time of protocol in the trained group in the bodily pain dimension.

## Data Availability

Data of the manuscript are available in Excel and Statistical program and can be shared online.
